# Consolidating Birth-Death and Death-Birth Processes in Structured Populations

**DOI:** 10.1371/journal.pone.0054639

**Published:** 2013-01-28

**Authors:** Joshua Zukewich, Venu Kurella, Michael Doebeli, Christoph Hauert

**Affiliations:** 1 Department of Mathematics, University of British Columbia, Vancouver, British Columbia, Canada; 2 Department of Zoology, University of British Columbia, Vancouver, British Columbia, Canada; University of Zaragoza, Spain

## Abstract

Network models extend evolutionary game theory to settings with spatial or social structure and have provided key insights on the mechanisms underlying the evolution of cooperation. However, network models have also proven sensitive to seemingly small details of the model architecture. Here we investigate two popular biologically motivated models of evolution in finite populations: Death-Birth (DB) and Birth-Death (BD) processes. In both cases reproduction is proportional to fitness and death is random; the only difference is the order of the two events at each time step. Although superficially similar, under DB cooperation may be favoured in structured populations, while under BD it never is. This is especially troubling as natural populations do not follow a strict one birth then one death regimen (or vice versa); such constraints are introduced to make models more tractable. Whether structure can promote the evolution of cooperation should not hinge on a simplifying assumption. Here, we propose a mixed rule where in each time step DB is used with probability 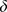 and BD is used with probability 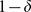. We derive the conditions for selection favouring cooperation under the mixed rule for all social dilemmas. We find that the only qualitatively different outcome occurs when using just BD (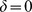). This case admits a natural interpretation in terms of kin competition counterbalancing the effect of kin selection. Finally we show that, for any mixed BD-DB update and under weak selection, cooperation is never inhibited by population structure for any social dilemma, including the Snowdrift Game.

## Introduction

Evolutionary game theory was developed to model frequency-dependent selection [1]. Arguably the most captivating system that exhibits frequency dependent selection is the evolution of cooperation under social dilemmas, a problem that has puzzled researchers across disciplines for decades (for a review, see [2]). In a social dilemma, cooperators provide a benefit to a group at some cost to self, while defectors pay no cost and contribute nothing. Groups of cooperators “do better” than groups of defectors, yet in any mixed group defectors “do best” [3]. The tension in social dilemmas is that defection maximizes a given individual's payoff while cooperation maximizes the total payoff to the group.

Network reciprocity is one of many approaches taken to explain the evolution of cooperation [4]. The network describes a spatially or socially structured population (see [5] for the first such example and [6] for a comprehensive review), in contrast to traditional evolutionary game theory, which assumes that populations are ‘well-mixed’ (where each individual interacts with every other individual with equal likelihood.) [7].

Evolution on networks is often modeled as a discrete-time birth-death process. Two popular updating rules (see e.g. [8]–[17]) are based on the frequency dependent Moran process [18]:

- *Birth-Death Update (BD):* At each update, an individual is chosen for reproduction with a probability proportional to its fitness; its offspring then replaces a randomly selected neighbour.
- *Death-Birth Update (DB):* At each update, an individual is chosen randomly for death; the vacant site is then filled by the offspring of one of its neighbours, selected with a probability proportional to fitness.

After many updates, eventually the finite population will be composed of only one type (in the absence of mutation), and we say that this type has reached fixation (further explanation in Section 1.1). Note that a well-mixed population (i.e. where individuals interact at random) can be described as a structured population where every individual is a neighbour of every other individual (the graph is “fully connected”) and so we may use these same update rules. For a structured population, the offspring of a parent is located close to the parent (called ‘limited dispersal’), which plays a crucial role in evolution in structured populations.

Ohtsuki *et al.* [8] found that spatial structure can promote the evolution of cooperation under DB, but not under the superficially similar rule, BD. In a complementary inclusive fitness approach, Taylor *et al.* [17] expressed the same disparity in results between BD and DB. In both rules reproduction is proportional to fitness and death is random, and yet they yield qualitatively different dynamics. This disparity is the focus of the present treatment.

In order to investigate the differences between BD and DB with limited dispersal, we introduce a parameter 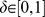 that allows a smooth transition between the two updating rules. Specifically, in each updating step we use DB with probability 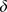 and BD with probability 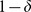. For 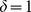 this results in pure DB dynamics, whereas for 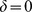 this results in pure BD dynamics. By varying 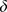 we can then identify qualitative changes in the evolutionary dynamics. As we are interested in the effect of the update rule, all simulations are carried out on the same network architecture: a simple square lattice.

### 1.1 The Moran Process

To model evolution in finite populations we use the Moran process [18], [19]. In each discrete time step one birth and one death occur, so the population size, 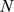, is constant. This assumes that ecological dynamics have come to a steady state in order to focus on evolutionary dynamics.

We consider the evolution of a population with two strategies, 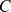 and 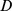. The state of the population is the number of 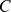 players, 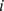 (and 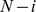 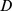-players). The Moran process is defined by the transition probabilities to go from 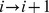 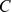-players (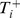) and from 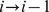 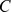-players (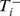). These probabilities depend on how likely it is to interact with either type (using the variable 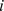 and information about the population structure) and the fitness effects that result from these interactions (using the parameters from the payoff matrix and the selection strength parameter, 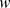).

We define fitnesses as a baseline fitness of 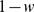 plus the payoff an individual receives weighted by 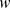. If 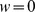, interactions have no effect on fitness and evolution is ‘neutral.’ Under neutral selection 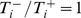 (the transition probabilities no longer depend on the payoffs). If interactions only have a small effect on fitness, selection is said to be ‘weak’ (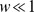), and 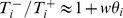, where we have introduced the coefficient 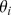 that captures the effects of population structure and update rules.

The quantity of interest in finite populations is the probability that a single 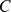 eventually replaces a resident 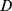 population (or the converse). This is termed the fixation probability of 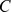, 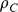 (or conversely, 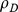). The fixation probabilities can be calculated as [18]:
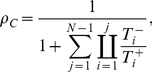
(1)

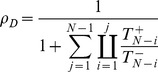
(2)


Under weak selection (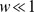 and 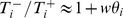), by Taylor expanding Eq. (1) –(2) in 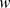 and ignoring higher order terms we find:
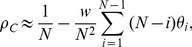
(3)

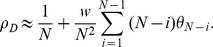
(4)


In the neutral case, in which 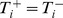, we have 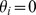, and hence both fixation probabilities are 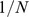. We say that 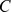 is a beneficial mutation (or simply beneficial) if 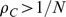, and 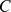 is detrimental if 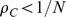. Using Eq. (3) and Eq. (4) , the conditions that 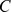 and 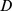 are beneficial are:
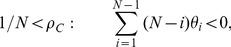
(5)

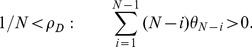
(6)


However, for some payoff matrices both 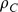 and 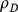 can simultaneously be less than 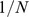 or greater than 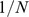. In these cases we say that 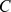 is favoured over 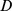 (or simply favoured) if 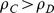. For example, if 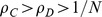, 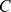 and 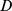 are both beneficial but 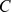 is favoured; or if 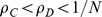, 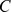 and 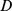 are both detrimental but 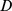 is favoured. To simplify the condition 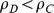 we first note that (2) can be rewritten as: 
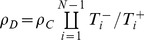
. Then, using 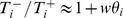 and ignoring higher order terms we find:
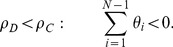
(7)


## Analysis

### 2.1 Structured Populations

In a structured population an individual's fitness depends on who its neighbours are. The transition probabilities depend not only on the number of 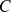
's in the population but also on the detailed configuration of the population – an unwieldy amount of data. To simplify, we first use Pair Approximation (see Appendix S1). The local density of 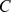
's around a 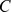 is 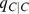 and indicates the conditional probability that the neighbour of a 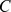 is another 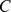. Similarly, 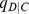 is the conditional probability of finding a 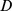 next to a 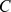, and so forth. Finally, 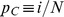 denotes the global frequency of 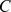
's.

The exact type of network is not of central importance in this treatment. For our analysis we assume all individuals have the same number of neighbours (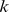 is a constant, and the network is “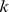-regular”). The Pair Approximation method used in our analysis is exact for infinite trees with no loops or leaves. All simulations are carried out on a square lattice (with 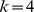); nevertheless, they agree well with our analytical approximations, despite the simulated networks being small and containing many loops.

In structured populations the weak selection limit (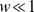) leads to a separation of timescales (see Appendix S1). In the initial phase of the population dynamics the local densities (e.g. 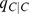) change quickly while the global densities (e.g. 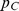) remain approximately constant. In a relatively short period, local densities reach a quasi-steady state where individuals are more likely to be surrounded by others of the same type. We can solve for this quasi-steady state by assuming that 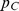 is constant and finding solutions to 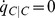; i.e. where the local densities are no longer changing (see Appendix S1, Eq. S1.5). This leads to the quasi-steady state solution:
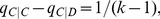
(8)


which means that, on average, a 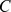 has 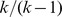 more 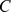
's in its neighborhood than a 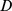 has in its neighborhood. Once the quasi-steady state is reached, the next phase of the population dynamics proceeds much slower. Gradually, the global densities change while local densities approximately satisfy the quasi-steady state solution, until eventually one type is lost completely (see Appendix S1).

### 2.1.1 Birth-Death (BD)

For BD updating, an individual is first selected from the population to reproduce with a probability proportional to fitness, and its offspring then replaces a random neighbour. To find the average fitness of a focal individual we condition on the number of cooperator neighbours it has. Let the focal individual have 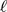 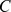-neighbours and 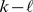 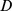-neighbours. If the focal individual is a cooperator, for instance, then it would have a fitness of:
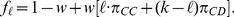
(9)


The 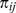 are the payoffs a type-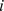 gets from each interaction with a type-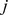. The average fitness of a cooperator is an average over all possible neighbourhoods, which is:
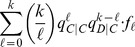
(10)


To probability that 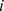 increases by one in a time step (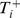) is the probability that a 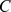 reproduces and a 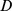 then dies, which is:
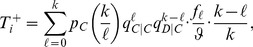
(11)


where 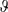 denotes the total fitness of all individuals in the population, which normalizes the fitnesses in order to use them probabilistically. The first factor in each term of Eq. (11) is the probability of finding a focal 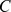 with a given neighbourhood; the second factor is its resulting relative fitness; and the third factor is the probability that the offspring replaces a 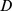 since death occurs uniformly randomly. Similarly (using 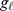 for the fitness of a defector with 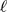 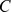-neighbours), we find (see Appendix S2):
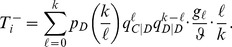
(12)


Recall that the ratio of the above transition probabilities (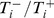) is the crucial quantity to determine the fixation probability of C's and D's (see Eq. (1) and Eq. (2) ). In the limit of weak selection, 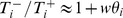 and it is this 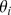 which tells us when cooperation is favoured, according to conditions (5)–(7). Here, we solve for 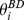 (see Appendix S2), as:
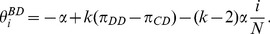
(13)


To get Eq. (13) we have used the quasi-steady state condition [ Eq. (8) ] and introduced 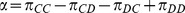 for convenience.

### 2.1.2 Death-Birth (DB)

For DB updating, one focal individual is randomly selected to die and its neighbours compete to fill the vacant site. The transition probabilities are again averages over the neighbourhoods of the focal individual:
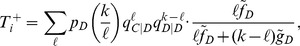
(14)

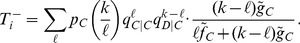
(15)


The first factor in each term of Eq. (14) [or Eq. (15) ] is the probability of finding the focal player (which is selected for death) in each arrangement and the second is the probability that it gets replaced by the opposite type. The 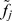 and 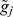 are the fitness of 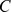 and 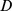 *neighbours* of a focal 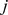 individual (see Appendix S2).

In the weak selection limit, simple expressions for 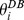 are analytically accessible. These determine whether cooperation is favoured (see Eq. (5) –(7)). We take the ratio of 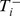 and 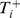 and expand as 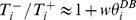, where
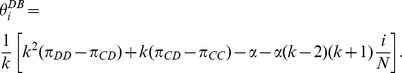
(16)


Again, we have used the quasi-steady state condition [ Eq. (8) ].

### 2.1.3 Mixed DB-BD Update

Under the mixed DB-BD rule (DB is used with probability 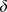 and BD with 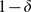) we find that the structural coefficient, 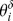, is a weighted average of 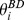 and 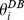 (see Appendix S2):
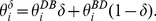
(17)


This result holds in the limit of weak selection (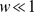). In this limit, any mixed update rule behaves the same to zero-th order in 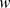; it is only the first order term where differences arise. Using 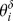 in conditions (5)–(7) determines whether 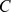 or 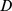 are beneficial mutations.

### 2.2 Well-Mixed Populations

For contrast and comparison we include the analyses of well-mixed populations under BD and DB. In a population with 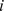 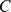-players, the fitness of a 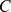-player (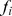) and a 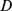-player (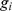) are:
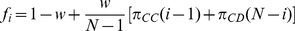
(18)

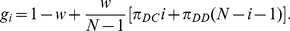
(19)


### 2.2.1 Birth-Death (BD)

For BD updating the ratio of the transition probabilities simplifies to: 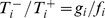 [18], which is approximately 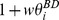 for 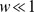, where:
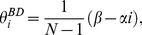
(20)


with 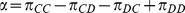 as before and 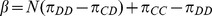 (see Appendix S2).

To find when 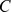 and 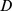 are favoured and beneficial, we insert Eq. (20) into conditions (5)–(7) and get (to leading order in 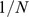 and 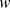):
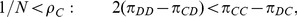
(21)

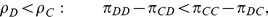
(22)

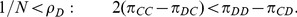
(23)


### 2.2.2 Death-Birth (DB)

Under DB the individual chosen for death cannot reproduce, and hence the ratio of the transition probabilities is slightly different than under BD updating. We find (see Appendix S2):
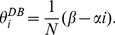
(24)


Note that 
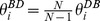
, and so the difference between BD and DB in well-mixed populations is negligible in large populations. Substituting into conditions (5)–(7) we find the same result as for BD. The only difference is the deviation from neutral selection, 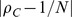, which is larger when using BD rather than DB.

In both cases birth is affected by fitness whereas death is uniformly random. In each BD time step an individual with high fitness may be selected for reproduction before it risks being killed. Under DB, an individual with high fitness could be selected to die before it ever has a chance of being selected for reproduction. Because the random step occurs before the step affected by selection, the noise in DB exceeds that in BD - and hence the fixation probabilities under DB are closer to those of an entirely random process, i.e. to 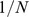.

### 2.3 Applications: Cooperation Games

Up to this point the types 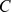 and 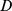 have been arbitrary labels, but the vast majority of game theory has been developed for social dilemmas between cooperators (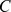) and defectors (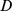). Social dilemmas are characterized by: (i) two 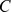
's do better than two 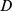
's (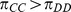); (ii) interacting with a 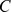 is always preferable to interacting with a 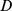 (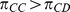 and 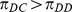); and finally (iii) a 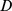 does better than a 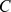 when they interact (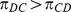). These restrictions leave four possible orderings of the payoffs [20]:
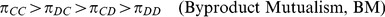
(25)

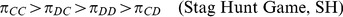
(26)

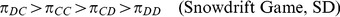
(27)

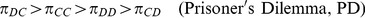
(28)


with popular names of the games in parentheses. Note that there is no ‘dilemma’ in BM games since cooperation is trivially favoured.

Here, we use a two-player version of the game in Hauert *et al.* [20]: Cooperators pay a cost 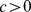 to provide a benefit 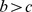 to a common pool, which will be equally split between the two players regardless of their strategies. Defectors pay no cost and contribute no benefit. In addition, we let the benefits be non-additive: the first contribution has weight one and the second has weight 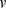. For 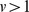 accumulated benefits are synergistically enhanced, whereas for 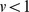 the benefit from the additional cooperator is discounted. The payoff matrix for the row player is:
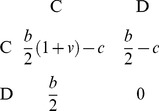
(29)


We normalize the payoff matrix by adding 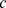, then dividing by 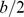. Note that in the Moran process adding a (positive) constant to the payoff matrix essentially reduces the selection strength. Similarly, dividing the payoffs by a factor larger than one further reduces selection strength. Since we are focussing on the weak selection limit, both rescaling operations are unproblematic as long as 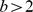 holds. After rescaling, the payoff matrix is:
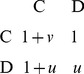
(30)


where 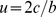 is the adjusted cost-benefit ratio. This payoff matrix encompasses all four social dilemmas: (i) 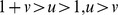 Prisoner's Dilemma; (ii) 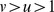 Stag-Hunt Game; (iii) 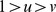 Snowdrift Game; and (iv) 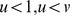 Byproduct Mutualism. Note, however, that if 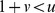 then we no longer have that two cooperators do better than two defectors (and so the game is not a “social dilemma”). The resulting game is Deadlock Defection, so called because there is no reason to ever cooperate. Byproduct Mutualism could similarly be termed Deadlock Cooperation.

## Results

**Figure 1:**
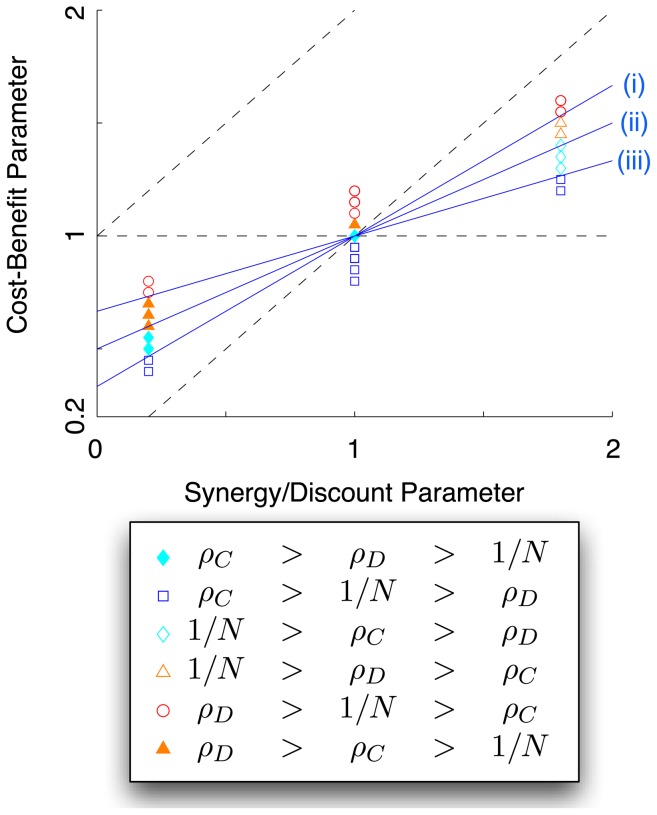
Favoured and beneficial strategies in social dilemmas for well-mixed populations. Parameter space of social dilemmas in well-mixed populations with the cost-to-benefit ratio 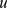 as the 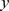-axis and the synergy/discounting parameter 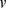 as the 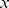-axis (see Eq. (30) ). The dashed lines divide the plane into five regions, which correspond to the Prisoner's Dilemma (PD), Snowdrift Game (SD), Stag-Hunt Game (SH), Deadlock Defection (DD) and Byproduct Mutualism (BM). The three solid lines are predictions for (i) 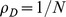 - above this line defection is beneficial; (ii) 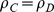 - below this line cooperation is favoured; and (iii) 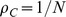 - below this line cooperation is beneficial. The three lines intersect at 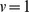; for 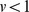, cooperation and defection may be simultaneously beneficial, while for 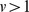 cooperation and defection may both be detrimental. Each data point represents simulation results for 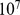 invasion attempts by a single cooperator and 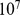 invasion attempts by a single defector. Parameters are: selection strength 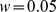, population size 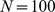.

**Figure 2:**
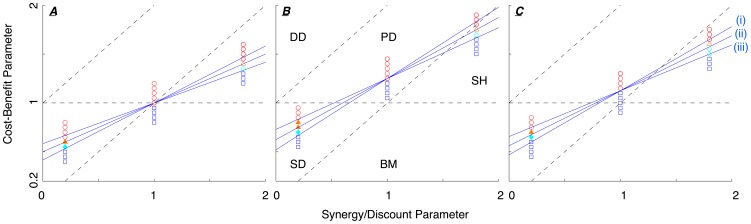
Favoured and beneficial strategies in social dilemmas for structured populations. Parameter space of social dilemmas in structured populations for BD updates (Panel ***A***), DB updates (Panel ***B***), and mixed BD-DB updates (Panel ***C***). The three solid lines indicate asymptotic predictions based on Pair Approximation for (i) 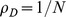, (ii) 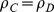, and (iii) 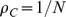. In Panel ***C***, DB and BD updates are chosen with equal chances (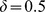). The black bolded triangle (Panels ***B*** and ***C***) indicates the parameter region where cooperators are favoured in Prisoner's Dilemmas. Parameter space organized as in Figure 1. Population structure is a lattice with connectivity 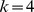. The simulation results (for 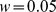 and 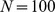), show good agreement with the analytical predictions (for 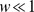 and 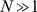).

The conditions for selection favouring 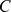 or 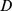 and for 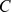 or 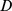 mutations being beneficial are summarized in Table 1 and Figures 1–2 for well-mixed and structured populations under BD, DB and mixed update rules. Table 1 illustrates that taking 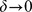 or 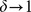 for the mixed rule recovers the results for the BD and DB updates. The limit 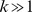 recovers the results for well-mixed populations - a highly connected population behaves like a well-mixed one.

**Table 1:** Favoured/beneficial strategies in social dilemmas under different structures/updates.

Condition:	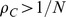	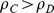	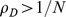
Well-Mixed	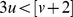	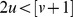	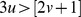
Spatial BD	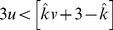	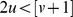	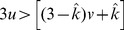
Spatial DB	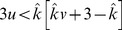	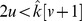	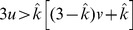
Spatial BD-DB	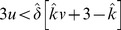	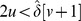	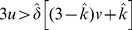

Conditions for 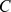 or 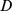 being beneficial (columns 2 and 4, respectively) and the condition for 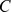 being favoured over 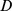 (column 3). Note that BD and DB yield the same conditions in well-mixed populations. 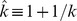 and 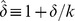 are used for convenience. Note: 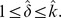

sssssIn the prisoner's dilemma (PD) cooperators are favoured (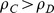) on a triangle in the 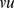-plane with vertices: 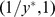, 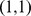, and 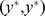, where 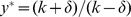 (bold black bordered in Fig. 2 Panel ***B***). This triangle has area 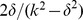 which grows with increasing 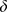 (see Fig. 2 Panel ***B*** for 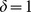) or decreasing 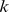. Hence, cooperation is enhanced for smaller neighbourhood sizes and when increasing the proportion of DB updates. In highly connected populations (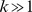) the triangle gets asymptotically small and disappears for well-mixed populations (see Fig. 1). The same happens when increasing the proportion of BD updates, and the triangle disappears for 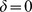 (see Fig. 2 Panel ***A***).

**Figure 3:**
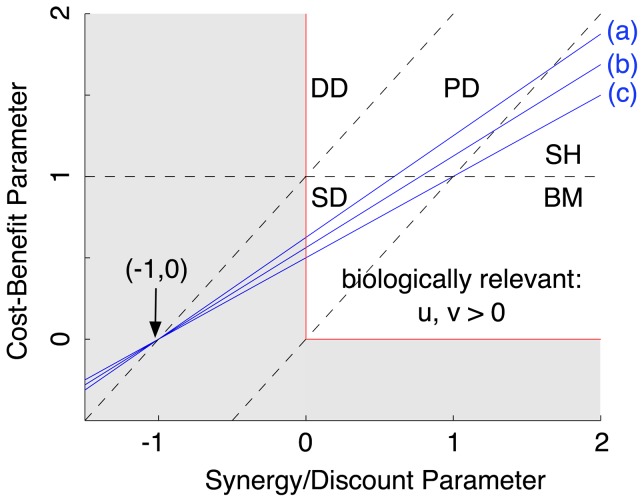
Comparison of update mechanisms in social dilemmas for structured populations. Parameter regions for cooperation in social dilemmas: predictions for well-mixed versus structured populations. The solid blue lines indicate predictions for 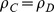 under different updates: (a) DB, (b) mixed BD-DB for 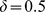, and (c) BD in structured populations. In well-mixed populations the condition is the same as (c). Population structure can extend the parameter region where cooperation is favored. The shaded area marks the extended parameter region, which has no biological interpretation in this framework.

A comparison of 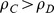 in the 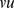-plane is displayed in Fig. 3 for different scenarios. Note that the parameter 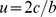 must be positive, since the costs and benefits must be positive to carry biological relevance. Similarly, additional benefits must remain beneficial, and so the synergy discount parameter 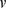 (which provides a weight for additional benefits) must also be positive. Whenever cooperators are favoured in well-mixed populations (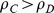), they are also favoured in structured populations (so long as 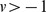). Thus, in the limit of weak selection, structure never inhibits cooperation in social dilemmas.

The critical lines (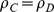) for structured and well-mixed populations intersect at 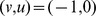 for any value of 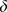. Within the range of biologically meaningful 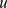, 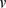, structure always promotes the evolution of cooperation. More generally, the conditions for 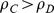 are:
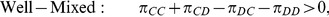
(31)

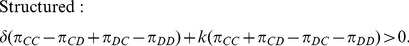
(32)


Social dilemmas require 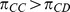 and 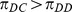, and hence if condition (31) is satisfied then so is condition (32), but the converse is not true in general.

The condition 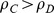 for structured populations under BD is the same as for well-mixed populations, reaffirming that 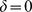 is a critical value for the evolutionary dynamics. Increasing the proportion of DB updates (increasing 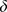) always makes cooperation more likely to evolve, at least in the limit of weak selection.

## Discussion and Conclusion

We set out to resolve the disparity between Birth-Death and Death-Birth updates for structured populations demonstrated by Ohtsuki *et al.* [8]. They considered a simple Prisoner's Dilemma where cooperators pay a cost 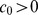 to donate a benefit 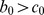 (subscripts used to distinguish from the 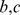 used in this paper) to their interaction partner and defectors neither provide benefits nor suffer costs. Under weak selection, they showed that cooperation is favoured and beneficial under the DB update if 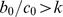, where 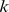 indicates the average number of neighbours. However, cooperation is never favoured or beneficial under BD.

Here, we introduced a mixed rule where at each time step DB is used with probability 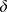 and BD with probability 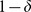. This allowed us to investigate the cause for the qualitative change in the evolutionary dynamics. To compare with Ohtsuki's work we can substitute their payoff matrix into equations (13), (16), (17) and (5)–(7). For our mixed BD-DB updating and weak selection the conditions for cooperators being beneficial and favoured all simplify to:
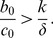
(33)


The condition for DB is recovered for 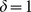 whereas 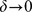 recovers the result for BD. The only qualitatively different outcome on the continuum between BD and DB occurs when using exclusively BD (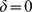). This suggests that in general, results based on BD updating may not be robust to small changes in the updating procedure. For any 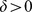, there is a critical cost-to-benefit ratio above which cooperation is favoured. This shows that the success of cooperators does not hinge on the sequence of events particular to DB, but is a more general phenomenon.

Spatial models capture the effect of limited dispersal, one consequence of which is that individuals are more likely to interact more with others of the same type (called positive assortment) than they would be in well-mixed populations. Positive assortment has a two-fold effect on populations facing social dilemmas: (i) cooperators may achieve a higher fitness through their interactions with other cooperators, but (ii) this increased fitness may be for naught if cooperators just replace other cooperators. (i) has often been called ‘kin selection’, while (ii) has been termed ‘kin competition,’ a distinction introduced by Hamilton [21]. Later work by Taylor *et al.* [22] showed that (i) and (ii) always balance in patch structured populations and hence altruism cannot evolve. However, this balancing does not necessarily occur in network-structured populations (e.g. [5], [8]).

Kin selection and competition provide an intuitive framework to understand the differences between BD and DB. Under both rules the fitness of all individuals is calculated before births and deaths and so the effect of kin selection does not depend on whether BD or DB is used. The difference then must lie in kin competition. Under DB, each individual has a 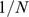 chance to die in each time step and so there is no effect of kin competition. Under BD, the likelihood of an individual dying depends on the fitnesses of its neighbours - the more fit its neighbours are, the more likely the focal individual will be replaced. This means that cooperators, who provide benefits to their neighbours, are actively increasing their own mortality.

Under BD the two effects of kin selection and competition exactly counterbalance. This balance represents a critical point in the evolutionary dynamics. Results derived at such a critical point are not robust. The mixed BD-DB update allows variable strength of kin competition: increasing 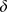 decreases kin competition. However, having kin competition outweigh kin selection would require further extension of the model - for instance by letting the interaction and replacement networks be different [10].

Our results fit cleanly within the framework introduced by Tarnita *et al.* [15]. They studied evolution in structured populations by defining a parameter 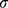 that captures the effect of structure and determines which type is favoured in the limit of weak selection. Under our assumptions, we find for the mixed BD-DB update that 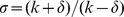. To get the 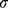 values for BD and DB in Tarnita *et al.* [15] we simply set 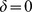 or 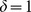, respectively. Again, it is apparent that 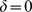 is a critical value as this implies 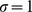: where structure has no effect on strategy selection [15].

The differences between BD and DB stem from a different balance of two biological effects of spatial structure. For BD, regions of the population occupied by high fitness individuals are updated more frequently than the vicinity of low fitness individuals. In contrast, for DB all regions are updated at equal rates. The mixed BD-DB update provides a transition between the two extremes and highlights that conclusions based on the BD update may not be robust.

Finally, we have shown that for the mixed BD-DB update, structure never inhibits the evolution of cooperation. For other update rules, or with strong selection, spatial structure may be detrimental to cooperation, e.g. in the Snowdrift Game [23]–[25]. This highlights the fact that results for structured populations should be explored for robustness to changes in model architecture.

## Supporting Information

Appendix S1. **Evolutionary Games on Graphs and Pair Approximation.**
(PDF)

Appendix S2. **Ratio of Transition Probabilities for Weak Selection.**
(PDF)
