# Supplementary material for: Consolidating Birth-Death and Death-Birth Processes in Structured Populations
Source: PLoS One. 2013 Jan 28;8(1):e54639. doi: 10.1371/journal.pone.0054639 (PMC3557300; doi:10.1371/journal.pone.0054639)
Supplement: Appendix S1. — Evolutionary Games on Graphs and Pair Approximation. (PDF) [file pone.0054639.s001.pdf]

Appendix S1: Evolutionary Games on Graphs and Pair Approximation

J. Zukewich[1*], V. Kurella[1], M. Doebeli[1,2], C. Hauert[1]

[1] Department of Mathematics, University of British Columbia, Vancouver, BC, Canada.
[2] Department of Zoology, University of British Columbia, Vancouver, BC, Canada.
∗ E-mail: Corresponding jzuke@math.ubc.ca

We model population structure by assigning individuals to the nodes of a graph where edges indicate their interaction partners. Each individual is connected to exactly k neighbours, forming a k-regular graph. The fitness of an individual depends on its neighbours, and so the transition probabilities (T_i^+ and T_i^-) depend on the configuration of the entire population.

In order to simplify the transition probabilities we use Pair Approximation [1, 2], which reduces the complex information about structure to information about pairs of individuals. Formally, let $q_{m|n}$ be the conditional probability of finding an m-type in the neighbourhood of a focal n-type. Pair Approximation then states that $q_{m|n\ell} = q_{m|n}$: the conditional probability that a neighbor of the focal n individual is of type m does not depend on any other type ℓ individual connected to the n. Pair Approximation is the most common approach, but see [3] and [2] for use of triplets or [4], [5] for n-point approximations ($n \leq 6$), and [6] for a numerical approach to improve Pair Approximation.

Pair Approximation involves two probabilities (p_C and p_D) and four conditional probabilities ($q_{C|C}$, $q_{D|C}$, $q_{C|D}$, $q_{D|D}$) plus four conserved quantities: $p_C + p_D = 1$, $q_{C|C} + q_{D|C} = 1$, $q_{D|D} + q_{C|D} = 1$ and $q_{D|C} p_C = q_{C|D} p_D$. The last equation follows from the fact that the number of $C - D$ edges must be the same as the number of $D - C$ edges. Hence, we have two free variables. We choose the global and local frequency of cooperators p_C and $q_{C|C}$. The expected change per time step in p_C is:

$$\frac{E(\Delta p_C)}{\Delta t} = \frac{T_i^+}{N} - \frac{T_i^-}{N} \approx -w \frac{T_i^+ \theta_i}{N}, \tag{S1.1}$$

where we have assumed $w \ll 1$ such that $T_i^- \approx T_i^+(1 + w\theta_i)$. Note that the $T_i^{\pm}$ depend not only on i (or, more precisely Np_C) but on $q_{C|C}$ as well. To find the expected change in $q_{C|C}$ per time step, we determine the number of $C - C$ links that are created or destroyed when a C replaces a D or vice versa. The total number of $C - C$ links is $q_{C|C} p_C \cdot (Nk/2)$ (note: there are $Nk/2$ total links in the population). If a C replaces a D, then the number of $C - C$ links increases by $1 + (k-1)q_{C|D}$. If a D replaces a C, then the number of $C - C$ links decreases by $(k-1)q_{C|C}$. The expected change in the number of $C - C$ links is:

$$\frac{Nk}{2} \frac{E(\Delta(q_{C|C} \cdot p_C))}{\Delta t} = T_i^+ \left[1 + (k-1)q_{C|D}\right] - T_i^- [(k-1)q_{C|C}]. \tag{S1.2}$$

Using $T_i^- \approx T_i^+(1 + w\theta_i)$, we have:

$$\frac{E(\Delta(q_{C|C} \cdot p_C))}{\Delta t} \approx \frac{2T_i^+}{Nk} \left[1 - (k-1)(q_{C|C} - q_{C|D})\right] + \mathcal{O}(w). \tag{S1.3}$$

Now we can expand $\Delta(q_{C|C} \cdot p_C) = q_{C|C} \Delta p_C + p_C \Delta q_{C|C} + \Delta p_C \Delta q_{C|C} = p_C \Delta q_{C|C} + \mathcal{O}(w)$ because Δp_C is $\mathcal{O}(w)$ (Eq. (S1.1)). Then we solve for $\Delta q_{C|C}$ as:

$$\frac{E(\Delta q_{C|C})}{\Delta t} \approx \frac{2T_i^+}{Nkp_C} \left[1 - (k-1)(q_{C|C} - q_{C|D})\right] + \mathcal{O}(w). \tag{S1.4}$$

$q_{C|C}$ changes fast ($\mathcal{O}(1)$, see Eq. (S1.4)) relative to p_C ($\mathcal{O}(w)$, see Eq. (S1.1)). Hence, we let the fast variable, $q_{C|C}$, go to its quasi-steady-state while the slow variable, p_C, stays approximately constant [7].

The quasi-steady state is satisfied when:

$$q_{C|C} - q_{C|D} = \frac{1}{k-1}. \qquad \text{(S1.5)}$$

Eq. (S1.5) (Eq. (8) in the main text) states that the updating mechanism leads to more C's being around a focal C than there are around a focal D. Population structure and limited dispersal provide positive assortment of types.

We take Eq. (S1.5) to always be satisfied and use it to simplify the transition probabilities. Once we choose a particular update mechanism, the transition probabilities become a function of just the number of C-players (as in the well-mixed case) and we can then use Eqs. (5)-(7) to find when C and D are beneficial and when C is favoured over D.

References

1. Matsuda H, Ogita N, Sasaki A, Sato K (1992) Statistical mechanics of population. Prog Theor Phys 88: 1035-1049.

2. Van Baalen M (2000) Pair approximations for different spatial geometries. In: Dieckmann U, Law R, Metz J, editors, The Geometry of Ecological Interactions: Simplifying Spatial Complexity, New York, NY, USA: Cambridge University Press.

3. Morita S (2008) Extended pair approximation of evolutionary game on complex networks. Prog Theor Phys 119: 29-38.

4. Szabó G, Tőke C (1998) Evolutionary prisoner's dilemma game on a square lattice. Phys Rev E 58: 69–73.

5. Szabó G, Antal T, Szabó P, Droz M (2000) Spatial evolutionary prisoner's dilemma game with three strategies and external constraints. Phys Rev E 62: 1095–1103.

6. Fu F, Wang L, Nowak M, Hauert C (2009) Evolutionary dynamics on graphs: Efficient method for weak selection. Phys Rev E 79: 046707.

7. Ohtsuki H, Hauert C, Lieberman E, Nowak M (2006) A simple rule for the evolution of cooperation on graphs. Nature 441: 502-505.
