# Supplementary material for: Consolidating Birth-Death and Death-Birth Processes in Structured Populations
Source: PLoS One. 2013 Jan 28;8(1):e54639. doi: 10.1371/journal.pone.0054639 (PMC3557300; doi:10.1371/journal.pone.0054639)
Supplement: Appendix S2. — Ratio of Transition Probabilities for Weak Selection. (PDF) [file pone.0054639.s002.pdf]

Appendix S2: Ratio of Transition Probabilities for Weak Selection

J. Zukewich[1*], V. Kurella[1], M. Doebeli[1,2], C. Hauert[1]

[1] Department of Mathematics, University of British Columbia, Vancouver, BC, Canada.
[2] Department of Zoology, University of British Columbia, Vancouver, BC, Canada.
∗ E-mail: Corresponding jzuke@math.ubc.ca

For each update rule and structure, the transition probabilities T_i^+, T_i^- are different. In each case we determine T_i^-/T_i^+ in the limit of weak selection ($w \ll 1$). In this limit $T_i^-/T_i^+ \approx 1 + w\theta_i$, where the coefficient θ_i captures the effect of population structure and update rules.

Well-Mixed Population (BD)

Under the BD rule the number of C's increases by one when a C reproduces and a D then dies:

$$T_i^+ = \frac{if_i}{if_i + (N-i)g_i}\frac{N-i}{N} \tag{S2.1}$$

Similarly, the number of C's decreases if a D reproduces and a C dies:

$$T_i^- = \frac{(N-i)g_i}{if_i + (N-i)g_i}\frac{i}{N} \tag{S2.2}$$

Hence $T_i^-/T_i^+ = g_i/f_i$, where $f_i = 1 - w + w[(i-1)\pi_{CC}) + (N-i)\pi_{CD}]$ and $g_i = 1 - w + w[i\pi_{DC} + (N - i - 1)\pi_{DD}]$ are the fitness of C and D given that there are i C-individuals and $N - i$ D-individuals [1]. By Taylor expanding and neglecting higher order terms we get:

$$T_i^-/T_i^+ \approx 1 - w(f_i - g_i), \tag{S2.3}$$

and hence

$$\theta_i^{BD} = -\alpha i + N(\pi_{DD} - \pi_{CD}) + \pi_{CC} - \pi_{DD}. \tag{S2.4}$$

where $\alpha = \pi_{CC} - \pi_{CD} - \pi_{DC} + \pi_{DD}$ is used for convenience.

Well-Mixed Population (DB)

Under the DB rule the number of C's increases by one when a D dies and a C then reproduces:

$$T_i^+ = \frac{N-i}{N}\frac{if_i}{if_i + (N-i-1)g_i}. \tag{S2.5}$$

Similarly, the number of Cs decreases if a C dies and a D reproduces:

$$T_i^- = \frac{i}{N}\frac{(N-i)g_i}{(i-1)f_i + (N-i)g_i}. \tag{S2.6}$$

Hence

$$T_i^-/T_i^+ = \frac{g_i}{f_i}\frac{(i-1)f_i + (N-i)g_i}{if_i + (N-i-1)g_i}, \tag{S2.7}$$

or, up to first order,

$$T_i^-/T_i^+ = 1 - w(f_i - g_i)\left(\frac{N}{N-1}\right) \tag{S2.8}$$

and

$$\theta_i^{DB} = \frac{N}{N-1}\theta_i^{BD}. \tag{S2.9}$$

Structured Population (BD)

The transition probabilities under BD for structured populations are given in Eqs. (9)-(10) based on the fitness of a focal cooperator (f_{k_C}) and a focal defector (g_{k_C}) with k_C C-neighbours:

$$f_{k_C} = 1 - w + w(k_C\pi_{CC} + (k - k_C)\pi_{CD}), \quad \text{(S2.10)}$$

$$g_{k_C} = 1 - w + w(k_C\pi_{DC} + (k - k_C)\pi_{DD}). \quad \text{(S2.11)}$$

In the limit of weak selection the separation of time scales results in the quasi-steady state condition (Eq. S1.5) that can be used to simplify Eqs. (9)-(10):

$$\frac{\phi T_i^+}{p_{CD}} = 1 + w[\pi_{CC} + \pi_{CD}(k-1) - 1 + (\pi_{CC} - \pi_{CD})(k-2)p_C], \quad \text{(S2.12)}$$

$$\frac{\phi T_i^-}{p_{CD}} = 1 + w[\pi_{DD} + \pi_{DC}(k-1) - 1 + (\pi_{DD} - \pi_{DC})(k-2)p_D], \quad \text{(S2.13)}$$

where ϕ indicates the total fitness of all individuals in the population. Using $T_i^-/T_i^+ \approx 1 + w\theta_i$, we find:

$$\theta_i^{BD} = -\alpha + k(\pi_{DD} - \pi_{CD}) - (k-2)\alpha\frac{i}{N}, \quad \text{(S2.14)}$$

with $\alpha = \pi_{CC} - \pi_{CD} - \pi_{DC} + \pi_{DD}$.

Structured Population (DB)

Under DB the transition probabilities are given in Eqs. (12)-(13) where $\tilde{f}_j$ and $\tilde{g}_j$ denote the fitness of C and D *neighbours* of a focal j individual:

$$\tilde{f}_j = 1 - w + w\{[\delta_{jC} + (k-1)q_{C|C}]\pi_{CC} + [\delta_{jD} + (k-1)q_{D|C}]\pi_{CD}\}, \quad \text{(S2.15)}$$

$$\tilde{g}_j = 1 - w + w\{[\delta_{jC} + (k-1)q_{C|D}]\pi_{DC} + [\delta_{jD} + (k-1)q_{D|D}]\pi_{DD}\}, \quad \text{(S2.16)}$$

where $\delta_{j\ell} = 1$ if $j = \ell$ and $\delta_{j\ell} = 0$ otherwise.

Using the quasi steady-state condition (**??**), Eqs. (12)-(13) simplify to:

$$T_i^+ = p_{CD}\left(1 - \frac{w\xi_{CD}}{k}\left[k - 1 - (k-2)p_C\right]\right), \quad \text{(S2.17)}$$

$$T_i^- = p_{CD}\left(1 - \frac{w\xi_{DC}}{k}\left[k - 1 - (k-2)p_D\right]\right), \quad \text{(S2.18)}$$

with

$$\xi_{ij} = (k-2)p_i(\pi_{ji} - \pi_{jj} - \pi_{ii} + \pi_{ij}) + k(\pi_{jj} - \pi_{ij}) - \pi_{ii} + \pi_{ij}. \quad \text{(S2.19)}$$

Using $T_i^-/T_i^+ \approx 1 + w\theta_i$, we find:

$$\theta_i^{DB} = \frac{1}{k}\left[k^2(\pi_{DD} - \pi_{CD}) + k(\pi_{CD} - \pi_{CC}) - \alpha - \alpha(k-2)(k+1)\frac{i}{N}\right]. \quad \text{(S2.20)}$$

Structured Population (Mixed Update)

Under the mixed update, DB is used with probability δ and BD with probability $1-\delta$. The probabilities to increase or decrease the number of C players by one C are:

$$T_i^{+\delta} = T_i^{+BD}(1-\delta) + T_i^{+DB}\delta, \quad \text{(S2.21)}$$

$$T_i^{-\delta} = T_i^{-BD}(1-\delta) + T_i^{-DB}\delta. \quad \text{(S2.22)}$$

Therefore, $\theta_i^\delta = \theta_i^{BD}(1-\delta) + \theta_i^{DB}\delta$.

References

1. Nowak M, Sasaki A, Taylor C, Fudenberg D (2004) Emergence of cooperation and evolutionary stability in finite populations. Nature 428: 646–650.
